# 3-(4-Chloro­phen­yl)-5-phenyl-1,2,4-triazolo[3,4-*a*]isoquinoline

**DOI:** 10.1107/S1600536810013668

**Published:** 2010-04-17

**Authors:** F. Nawaz Khan, P. Manivel, K. Prabakaran, Venkatesha R. Hathwar, Mehmet Akkurt

**Affiliations:** a Organic and Medicinal Chemistry Research Laboratory, Organic Chemistry Division, School of Advanced Sciences, VIT University, Vellore 632 014, Tamil Nadu, India; b Solid State and Structural Chemistry Unit, Indian Institute of Science, Bangalore 560 012, Karnataka, India; c Department of Physics, Faculty of Arts and Sciences, Erciyes University, 38039 Kayseri, Turkey

## Abstract

In the title mol­ecule, C_22_H_14_ClN_3_, the triazoloisoquinoline ring system is approximately planar, with an r.m.s. deviation of 0.033 (2) Å and a maximum departure from the mean plane of 0.062 (1) Å for the triazole ring C atom, bonded to the benzene ring. The benzene and phenyl rings are twisted by 57.02 (6) and 62.16 (6)°, respectively, to the mean plane of the triazoloisoquinoline ring system. The mol­ecule is stabilized by a weak intra­molecular π–π inter­action [centroid–centroid distance = 3.7089 (10) Å] between the benzene and phenyl rings. In the crystal structure, weak inter­molecular C—H⋯N hydrogen bonds and C—H⋯π inter­actions link the mol­ecules.

## Related literature

For the synthesis and anti­helmintic activity of triazolo compounds similar to the title compound, see: Nadkarni *et al.* (2001 ▶); Hui *et al.* (1999 ▶). For related structures, see: Khan *et al.* (2010 ▶); Zou *et al.* (2004 ▶).
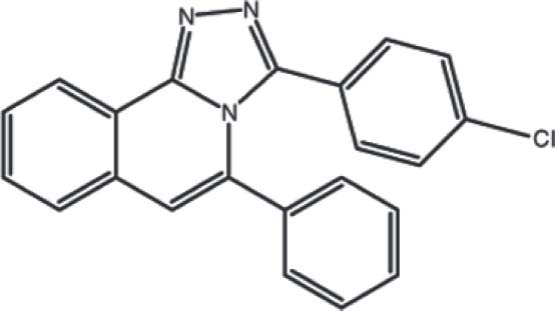


## Experimental

### 

#### Crystal data

C_22_H_14_ClN_3_
*M* *_r_* = 355.81
Monoclinic, 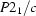

*a* = 7.9841 (3) Å
*b* = 9.0679 (4) Å
*c* = 23.9881 (11) Å
β = 93.078 (4)°
*V* = 1734.20 (13) Å^3^
*Z* = 4
Mo *K*α radiation
μ = 0.23 mm^−1^
*T* = 290 K
0.40 × 0.32 × 0.25 mm

#### Data collection

Oxford Xcalibur Eos (Nova) CCD detector diffractometer
Absorption correction: multi-scan (*CrysAlis PRO RED*; Oxford Diffraction, 2009 ▶) *T*_min_ = 0.902, *T*_max_ = 0.945
19413 measured reflections
3216 independent reflections
2090 reflections with *I* > 2σ(*I*)
*R*_int_ = 0.042

#### Refinement

*R*[*F* ^2^ > 2σ(*F* ^2^)] = 0.038
*wR*(*F* ^2^) = 0.097
*S* = 0.97
3216 reflections
235 parameters
H-atom parameters constrained
Δρ_max_ = 0.11 e Å^−3^
Δρ_min_ = −0.21 e Å^−3^

### 

Data collection: *CrysAlis PRO CCD* (Oxford Diffraction, 2009 ▶); cell refinement: *CrysAlis PRO CCD*; data reduction: *CrysAlis PRO RED* (Oxford Diffraction, 2009 ▶); program(s) used to solve structure: *SHELXS97* (Sheldrick, 2008 ▶); program(s) used to refine structure: *SHELXL97* (Sheldrick, 2008 ▶); molecular graphics: *ORTEP-3* (Farrugia, 1997 ▶); software used to prepare material for publication: *WinGX* (Farrugia, 1999 ▶).

## Supplementary Material

Crystal structure: contains datablocks global, I. DOI: 10.1107/S1600536810013668/bg2341sup1.cif

Structure factors: contains datablocks I. DOI: 10.1107/S1600536810013668/bg2341Isup2.hkl

Additional supplementary materials:  crystallographic information; 3D view; checkCIF report

## Figures and Tables

**Table 1:** Hydrogen-bond geometry (Å, °) *Cg*1, *Cg*2 and *Cg*3 are the centroids of the N1–N3/C1/C16, N1/C1/C2/C7–C9 and C2–C7 rings, respectively.

*D*—H⋯*A*	*D*—H	H⋯*A*	*D*⋯*A*	*D*—H⋯*A*
C6—H6⋯N2^i^	0.93	2.59	3.514 (2)	170
C8—H8⋯N3^i^	0.93	2.62	3.496 (2)	156
C18—H18⋯*Cg*1^ii^	0.93	2.70	3.4524 (17)	138
C21—H21⋯*Cg*3^iii^	0.93	2.89	3.7139 (19)	149
C22—H22⋯*Cg*2^iii^	0.93	2.90	3.5442 (18)	128

Symmetry codes: (i) 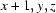; (ii) 
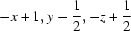
; (iii) 
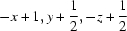
.

**Fig. 1.**
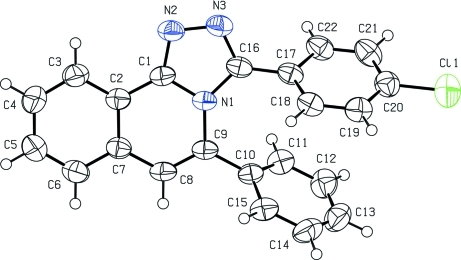
The title molecule with the atom numbering scheme. Displacement ellipsoids for non-H atoms are drawn at the 50% probability level.

**Fig. 2.**
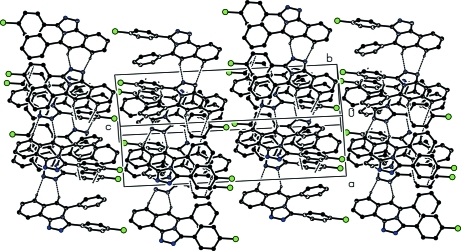
A generel view of the packing diagram and the hydrogen bonding of (I). H atoms not involved in the motif shown have been omitted for clarity.

## supplementary crystallographic information

### Comment

As part of our search for new isoquinoline analogues, we focused on the synthesis of the titled compound, which crystal structure is reported.

In the title molecule (I), Fig. 1, the triazoloisoquinoline ring system (N1–N3/C1–C9/C16) is approximately planar, with an r.m.s. deviation of 0.033 (2) Å and a maximum departure from the mean plane of -0.062 (1) Å for the triazole ring C16 atom, bonded to the benzene ring (C17–C22). The benzene (C17–C22) and phenyl (C10–C15) rings are twisted by 57.02 (6) and 62.16 (6) ° with respect to the mean plane of the triazoloisoquinoline ring system. The dihedral angle betwen the benzene (C17–C22) and phenyl (C10–C15) rings is 22.21 (8)° .

The molecule is stabilized by a weak intramolecular π-π interaction [*Cg*4···*Cg*5(*x*, *y*, *z*) = 3.7089 (10) Å; *Cg*4 and *Cg*5 are the centroids of the rings C10–C15 and C17–C22, respectively]. In the crystal structure, weak intermolecular C—H···N hydrogen bonds and C—H···π interactions (Table 1, Fig. 2) link the molecules to each other.

### Experimental

2-(3-Phenylisoquinolin-1-yl)hydrazine (1 mmol) was condensed with, 4-chlorobenzaldehyde (1.1 mmol) under refluxing conditions in isopropanol (10 ml) solvent to give the corresponding hydrazone in high yield. After removal of the solvent the compound was then oxidatively cyclized in nitrobenzene (10 ml) at 473 K. The product was recrystallized from dichlomethane to give block-shaped crystals.

### Refinement

H atoms were placed at calculated positions with C–H = 0.93 Å and were included in the refinement in the riding model approximation, with *U_iso_*(H) = 1.2*U_eq_*(C).

### Crystal data

**Table:**

C_22_H_14_ClN_3_	*F*(000) = 736
*M**_r_* = 355.81	*D*_x_ = 1.363 Mg m^−^^3^
Monoclinic, *P*2_1_/*c*	Mo *K*α radiation, λ = 0.71073 Å
Hall symbol: -P 2ybc	Cell parameters from 953 reflections
*a* = 7.9841 (3) Å	θ = 1.7–20.4°
*b* = 9.0679 (4) Å	µ = 0.23 mm^−^^1^
*c* = 23.9881 (11) Å	*T* = 290 K
β = 93.078 (4)°	Block, colourless
*V* = 1734.20 (13) Å^3^	0.40 × 0.32 × 0.25 mm
*Z* = 4	

### Data collection

**Table:**

Oxford Xcalibur Eos (Nova) CCD detector diffractometer	3216 independent reflections
Radiation source: Enhance (Mo) X-ray Source	2090 reflections with *I* > 2σ(*I*)
graphite	*R*_int_ = 0.042
ω scans	θ_max_ = 25.5°, θ_min_ = 3.0°
Absorption correction: multi-scan (*CrysAlis PRO* RED; Oxford Diffraction, 2009)	*h* = −9→9
*T*_min_ = 0.902, *T*_max_ = 0.945	*k* = −10→10
19413 measured reflections	*l* = −29→29

### Refinement

**Table:**

Refinement on *F*^2^	Primary atom site location: structure-invariant direct methods
Least-squares matrix: full	Secondary atom site location: difference Fourier map
*R*[*F*^2^ > 2σ(*F*^2^)] = 0.038	Hydrogen site location: inferred from neighbouring sites
*wR*(*F*^2^) = 0.097	H-atom parameters constrained
*S* = 0.97	*w* = 1/[σ^2^(*F*_o_^2^) + (0.0512*P*)^2^] where *P* = (*F*_o_^2^ + 2*F*_c_^2^)/3
3216 reflections	(Δ/σ)_max_ < 0.001
235 parameters	Δρ_max_ = 0.11 e Å^−^^3^
0 restraints	Δρ_min_ = −0.20 e Å^−^^3^

### Special details

**Table:**

Geometry. All esds (except the esd in the dihedral angle between two l.s. planes) are estimated using the full covariance matrix. The cell esds are taken into account individually in the estimation of esds in distances, angles and torsion angles; correlations between esds in cell parameters are only used when they are defined by crystal symmetry. An approximate (isotropic) treatment of cell esds is used for estimating esds involving l.s. planes.
Refinement. Refinement of *F*^2^ against ALL reflections. The weighted *R*-factor wR and goodness of fit *S* are based on *F*^2^, conventional *R*-factors *R* are based on *F*, with *F* set to zero for negative *F*^2^. The threshold expression of *F*^2^ > σ(*F*^2^) is used only for calculating *R*-factors(gt) etc. and is not relevant to the choice of reflections for refinement. *R*-factors based on *F*^2^ are statistically about twice as large as those based on *F*, and *R*- factors based on ALL data will be even larger.

### Fractional atomic coordinates and isotropic or equivalent isotropic displacement parameters (Å^2^)

**Table:**

	*x*	*y*	*z*	*U*_iso_*/*U*_eq_	
Cl1	0.31231 (8)	0.36657 (7)	0.47872 (2)	0.0881 (2)	
N1	0.59862 (15)	0.38872 (12)	0.21077 (5)	0.0397 (3)	
C9	0.76959 (18)	0.38731 (15)	0.23083 (7)	0.0420 (4)	
C7	0.84736 (19)	0.37494 (16)	0.13340 (7)	0.0438 (4)	
C8	0.8862 (2)	0.37965 (16)	0.19258 (7)	0.0465 (4)	
H8	0.9984	0.3773	0.2051	0.056*	
C2	0.67799 (19)	0.38170 (16)	0.11339 (7)	0.0428 (4)	
C1	0.55381 (19)	0.39096 (16)	0.15404 (7)	0.0421 (4)	
C10	0.81034 (19)	0.39579 (17)	0.29162 (7)	0.0434 (4)	
C17	0.41623 (19)	0.38678 (16)	0.29626 (7)	0.0438 (4)	
C16	0.44668 (19)	0.39497 (16)	0.23649 (7)	0.0435 (4)	
N2	0.38951 (16)	0.40120 (14)	0.14584 (6)	0.0515 (4)	
N3	0.32375 (16)	0.40415 (15)	0.19789 (6)	0.0514 (4)	
C6	0.9712 (2)	0.36397 (18)	0.09438 (8)	0.0555 (5)	
H6	1.0837	0.3616	0.1066	0.067*	
C20	0.3518 (2)	0.3739 (2)	0.40853 (7)	0.0543 (5)	
C18	0.4740 (2)	0.26909 (18)	0.32926 (7)	0.0484 (4)	
H18	0.5349	0.1940	0.3133	0.058*	
C3	0.6365 (2)	0.37670 (19)	0.05624 (8)	0.0577 (5)	
H3	0.5248	0.3823	0.0432	0.069*	
C19	0.4425 (2)	0.26216 (19)	0.38509 (7)	0.0536 (5)	
H19	0.4819	0.1832	0.4068	0.064*	
C21	0.2899 (2)	0.4902 (2)	0.37646 (8)	0.0608 (5)	
H21	0.2271	0.5640	0.3923	0.073*	
C5	0.9281 (2)	0.3568 (2)	0.03855 (8)	0.0668 (5)	
H5	1.0115	0.3473	0.0132	0.080*	
C11	0.7686 (2)	0.51875 (18)	0.32226 (7)	0.0519 (4)	
H11	0.7176	0.5991	0.3042	0.062*	
C22	0.3224 (2)	0.49554 (19)	0.32059 (8)	0.0541 (5)	
H22	0.2806	0.5735	0.2989	0.065*	
C15	0.8920 (2)	0.27925 (19)	0.31932 (7)	0.0552 (5)	
H15	0.9245	0.1971	0.2993	0.066*	
C12	0.8017 (2)	0.5234 (2)	0.37932 (8)	0.0627 (5)	
H12	0.7719	0.6062	0.3994	0.075*	
C4	0.7606 (3)	0.3635 (2)	0.01925 (8)	0.0705 (6)	
H4	0.7329	0.3591	−0.0189	0.085*	
C14	0.9250 (2)	0.2852 (2)	0.37642 (8)	0.0679 (6)	
H14	0.9791	0.2067	0.3947	0.081*	
C13	0.8784 (3)	0.4062 (2)	0.40646 (8)	0.0692 (6)	
H13	0.8988	0.4086	0.4450	0.083*	

### Atomic displacement parameters (Å^2^)

**Table:**

	*U*^11^	*U*^22^	*U*^33^	*U*^12^	*U*^13^	*U*^23^
Cl1	0.0944 (4)	0.1095 (5)	0.0616 (4)	0.0059 (3)	0.0163 (3)	0.0034 (3)
N1	0.0311 (7)	0.0376 (8)	0.0498 (8)	0.0006 (5)	−0.0026 (6)	−0.0017 (6)
C9	0.0333 (8)	0.0365 (9)	0.0552 (11)	−0.0003 (7)	−0.0060 (8)	−0.0025 (7)
C7	0.0389 (9)	0.0396 (10)	0.0527 (11)	−0.0010 (7)	−0.0007 (8)	−0.0034 (8)
C8	0.0324 (8)	0.0480 (10)	0.0579 (11)	0.0006 (7)	−0.0081 (8)	−0.0035 (8)
C2	0.0394 (9)	0.0379 (10)	0.0506 (11)	0.0012 (7)	−0.0031 (8)	−0.0051 (8)
C1	0.0369 (9)	0.0377 (10)	0.0507 (11)	0.0006 (7)	−0.0077 (8)	−0.0028 (7)
C10	0.0359 (8)	0.0429 (10)	0.0505 (10)	−0.0047 (7)	−0.0054 (7)	−0.0001 (8)
C17	0.0349 (8)	0.0376 (10)	0.0590 (11)	−0.0045 (7)	0.0047 (8)	0.0024 (8)
C16	0.0346 (8)	0.0368 (10)	0.0589 (11)	−0.0023 (7)	0.0011 (8)	0.0003 (8)
N2	0.0355 (8)	0.0589 (10)	0.0593 (10)	−0.0008 (6)	−0.0046 (7)	−0.0029 (7)
N3	0.0361 (7)	0.0562 (9)	0.0614 (10)	−0.0033 (6)	−0.0009 (7)	0.0018 (7)
C6	0.0410 (9)	0.0599 (12)	0.0654 (13)	0.0036 (8)	0.0017 (9)	−0.0061 (9)
C20	0.0513 (10)	0.0557 (12)	0.0565 (12)	−0.0062 (9)	0.0074 (9)	0.0003 (9)
C18	0.0453 (10)	0.0369 (10)	0.0634 (12)	0.0004 (7)	0.0058 (8)	−0.0005 (9)
C3	0.0483 (10)	0.0688 (13)	0.0549 (12)	0.0052 (9)	−0.0065 (9)	−0.0086 (9)
C19	0.0523 (11)	0.0455 (11)	0.0629 (12)	−0.0016 (8)	0.0012 (9)	0.0083 (9)
C21	0.0576 (11)	0.0516 (12)	0.0750 (14)	0.0062 (9)	0.0187 (10)	0.0001 (10)
C5	0.0571 (12)	0.0837 (14)	0.0604 (13)	0.0074 (10)	0.0111 (10)	−0.0074 (11)
C11	0.0538 (11)	0.0430 (10)	0.0582 (12)	−0.0032 (8)	−0.0030 (9)	−0.0006 (9)
C22	0.0489 (10)	0.0447 (11)	0.0695 (13)	0.0059 (8)	0.0103 (9)	0.0087 (9)
C15	0.0503 (11)	0.0506 (11)	0.0636 (12)	0.0043 (8)	−0.0075 (9)	0.0006 (9)
C12	0.0687 (12)	0.0608 (13)	0.0586 (13)	−0.0125 (10)	0.0018 (10)	−0.0137 (10)
C4	0.0704 (14)	0.0933 (16)	0.0473 (11)	0.0114 (11)	−0.0019 (10)	−0.0106 (10)
C14	0.0628 (12)	0.0744 (14)	0.0644 (14)	0.0050 (11)	−0.0154 (10)	0.0145 (11)
C13	0.0693 (13)	0.0853 (17)	0.0517 (12)	−0.0141 (12)	−0.0087 (10)	0.0011 (12)

### Geometric parameters (Å, °)

**Table:**

Cl1—C20	1.7307 (18)	C20—C21	1.381 (2)
N1—C1	1.3886 (19)	C20—C19	1.383 (2)
N1—C16	1.3913 (19)	C18—C19	1.377 (2)
N1—C9	1.4228 (18)	C18—H18	0.9300
C9—C8	1.343 (2)	C3—C4	1.371 (3)
C9—C10	1.479 (2)	C3—H3	0.9300
C7—C6	1.401 (2)	C19—H19	0.9300
C7—C2	1.412 (2)	C21—C22	1.380 (2)
C7—C8	1.437 (2)	C21—H21	0.9300
C8—H8	0.9300	C5—C4	1.393 (3)
C2—C3	1.394 (2)	C5—H5	0.9300
C2—C1	1.430 (2)	C11—C12	1.381 (2)
C1—N2	1.3193 (19)	C11—H11	0.9300
C10—C11	1.386 (2)	C22—H22	0.9300
C10—C15	1.392 (2)	C15—C14	1.382 (2)
C17—C22	1.386 (2)	C15—H15	0.9300
C17—C18	1.393 (2)	C12—C13	1.374 (3)
C17—C16	1.469 (2)	C12—H12	0.9300
C16—N3	1.315 (2)	C4—H4	0.9300
N2—N3	1.3804 (19)	C14—C13	1.375 (3)
C6—C5	1.366 (2)	C14—H14	0.9300
C6—H6	0.9300	C13—H13	0.9300
			
C1—N1—C16	104.42 (12)	C19—C18—H18	119.5
C1—N1—C9	121.57 (13)	C17—C18—H18	119.5
C16—N1—C9	133.95 (14)	C4—C3—C2	119.83 (17)
C8—C9—N1	117.18 (14)	C4—C3—H3	120.1
C8—C9—C10	123.53 (14)	C2—C3—H3	120.1
N1—C9—C10	119.28 (14)	C18—C19—C20	119.27 (16)
C6—C7—C2	118.25 (15)	C18—C19—H19	120.4
C6—C7—C8	122.66 (15)	C20—C19—H19	120.4
C2—C7—C8	119.09 (15)	C22—C21—C20	119.20 (17)
C9—C8—C7	123.76 (14)	C22—C21—H21	120.4
C9—C8—H8	118.1	C20—C21—H21	120.4
C7—C8—H8	118.1	C6—C5—C4	120.67 (18)
C3—C2—C7	120.39 (16)	C6—C5—H5	119.7
C3—C2—C1	122.38 (14)	C4—C5—H5	119.7
C7—C2—C1	117.21 (14)	C12—C11—C10	120.77 (16)
N2—C1—N1	110.42 (15)	C12—C11—H11	119.6
N2—C1—C2	128.52 (15)	C10—C11—H11	119.6
N1—C1—C2	121.06 (13)	C21—C22—C17	121.20 (16)
C11—C10—C15	118.54 (15)	C21—C22—H22	119.4
C11—C10—C9	121.21 (14)	C17—C22—H22	119.4
C15—C10—C9	120.25 (14)	C14—C15—C10	120.20 (17)
C22—C17—C18	118.43 (16)	C14—C15—H15	119.9
C22—C17—C16	119.77 (15)	C10—C15—H15	119.9
C18—C17—C16	121.76 (15)	C13—C12—C11	120.20 (18)
N3—C16—N1	109.02 (14)	C13—C12—H12	119.9
N3—C16—C17	122.28 (14)	C11—C12—H12	119.9
N1—C16—C17	128.65 (14)	C3—C4—C5	120.23 (18)
C1—N2—N3	106.85 (13)	C3—C4—H4	119.9
C16—N3—N2	109.26 (13)	C5—C4—H4	119.9
C5—C6—C7	120.60 (16)	C13—C14—C15	120.54 (17)
C5—C6—H6	119.7	C13—C14—H14	119.7
C7—C6—H6	119.7	C15—C14—H14	119.7
C21—C20—C19	120.84 (17)	C12—C13—C14	119.70 (18)
C21—C20—Cl1	119.56 (15)	C12—C13—H13	120.2
C19—C20—Cl1	119.60 (14)	C14—C13—H13	120.2
C19—C18—C17	121.02 (16)		
			
C1—N1—C9—C8	−3.74 (19)	C18—C17—C16—N1	−55.3 (2)
C16—N1—C9—C8	179.68 (14)	N1—C1—N2—N3	−0.67 (16)
C1—N1—C9—C10	175.62 (12)	C2—C1—N2—N3	178.84 (14)
C16—N1—C9—C10	−1.0 (2)	N1—C16—N3—N2	1.22 (16)
N1—C9—C8—C7	0.9 (2)	C17—C16—N3—N2	−176.41 (12)
C10—C9—C8—C7	−178.46 (13)	C1—N2—N3—C16	−0.35 (16)
C6—C7—C8—C9	−178.68 (15)	C2—C7—C6—C5	−1.4 (2)
C2—C7—C8—C9	1.4 (2)	C8—C7—C6—C5	178.64 (15)
C6—C7—C2—C3	0.3 (2)	C22—C17—C18—C19	−1.5 (2)
C8—C7—C2—C3	−179.71 (13)	C16—C17—C18—C19	−179.15 (14)
C6—C7—C2—C1	179.22 (14)	C7—C2—C3—C4	0.7 (2)
C8—C7—C2—C1	−0.8 (2)	C1—C2—C3—C4	−178.11 (16)
C16—N1—C1—N2	1.37 (15)	C17—C18—C19—C20	0.1 (2)
C9—N1—C1—N2	−176.09 (12)	C21—C20—C19—C18	1.3 (3)
C16—N1—C1—C2	−178.19 (13)	Cl1—C20—C19—C18	−179.73 (12)
C9—N1—C1—C2	4.4 (2)	C19—C20—C21—C22	−1.3 (3)
C3—C2—C1—N2	−2.6 (2)	Cl1—C20—C21—C22	179.77 (13)
C7—C2—C1—N2	178.59 (14)	C7—C6—C5—C4	1.5 (3)
C3—C2—C1—N1	176.91 (13)	C15—C10—C11—C12	−2.3 (2)
C7—C2—C1—N1	−1.9 (2)	C9—C10—C11—C12	177.63 (15)
C8—C9—C10—C11	116.02 (18)	C20—C21—C22—C17	−0.2 (3)
N1—C9—C10—C11	−63.30 (19)	C18—C17—C22—C21	1.6 (2)
C8—C9—C10—C15	−64.0 (2)	C16—C17—C22—C21	179.24 (15)
N1—C9—C10—C15	116.67 (16)	C11—C10—C15—C14	2.2 (2)
C1—N1—C16—N3	−1.56 (15)	C9—C10—C15—C14	−177.82 (15)
C9—N1—C16—N3	175.42 (14)	C10—C11—C12—C13	0.7 (3)
C1—N1—C16—C17	175.88 (14)	C2—C3—C4—C5	−0.7 (3)
C9—N1—C16—C17	−7.1 (2)	C6—C5—C4—C3	−0.4 (3)
C22—C17—C16—N3	−55.7 (2)	C10—C15—C14—C13	−0.3 (3)
C18—C17—C16—N3	121.87 (17)	C11—C12—C13—C14	1.2 (3)
C22—C17—C16—N1	127.15 (17)	C15—C14—C13—C12	−1.4 (3)

### Hydrogen-bond geometry (Å, °)

**Table:**

Cg1, Cg2 and Cg3 are the centroids of the N1–N3/C1/C16, N1/C1/C2/C7–C9 and C2–C7 rings, respectively.

**Table:**

*D*—H···*A*	*D*—H	H···*A*	*D*···*A*	*D*—H···*A*
C6—H6···N2^i^	0.93	2.59	3.514 (2)	170
C8—H8···N3^i^	0.93	2.62	3.496 (2)	156
C18—H18···Cg1^ii^	0.93	2.70	3.4524 (17)	138
C21—H21···Cg3^iii^	0.93	2.89	3.7139 (19)	149
C22—H22···Cg2^iii^	0.93	2.90	3.5442 (18)	128

Symmetry codes: (i) *x*+1, *y*, *z*; (ii) −*x*+1, *y*−1/2, −*z*+1/2; (iii) −*x*+1, *y*+1/2, −*z*+1/2.
